# Osteochondroma Involving the Ramus of the Mandible: An Unusual Location

**DOI:** 10.1155/2020/8603027

**Published:** 2020-11-25

**Authors:** Pakki Suresh Kumar, Dola Srinivasa Rao, Swapna Manepalli, Ajit Damera, Jai Kiran Killada

**Affiliations:** ^1^Department of Oral Medicine and Radiology, GITAM Dental College and Hospital, Visakhapatnam, Andhra Pradesh, India; ^2^Department of Periodontics, GITAM Dental College and Hospital, Visakhapatnam, Andhra Pradesh, India; ^3^Department of Paedodontics and Preventive Dentistry, GITAM Dental College, Visakhapatnam, India

## Abstract

Osteochondroma (OC) is considered the most common tumor of the axial skeleton, although it is relatively uncommon in the craniofacial region. The present case describes an atypical case of OC in the posterior border of the ramus of the mandible. To the best of our knowledge, only one case has been reported till now in this region. A 35-year-old male patient reported with a complaint of swelling over the left middle one-third of the face and limited mouth opening for six years. Panoramic radiograph (PR) and paranasal sinus (PNS) view showed a well-defined radiopacity located in this region. Computed tomography (CT) and magnetic resonance imaging (MRI) revealed a characteristic mushroom-shaped outgrowth from the inner surface of the ramus of the mandible. The patient was treated with osteotomy, with the cut made at the angle of the mandible. Histopathological examination revealed features suggestive of OC. Subsequently, the patient was able to open his mouth, and there was no evidence of recurrence or postoperative complications in the one-year follow-up.

## 1. Introduction

Osteochondroma (OC) is a primary benign bone tumor of the long bones commonly involving the axial skeleton such as the metaphysis of the femur and tibia bones [1]. OC of the craniofacial bones is extremely rare because the facial bones exhibit intramembranous ossification, whereas the tumor usually appears in endochondral bones [2]. To the best of our knowledge, the last 30 years revealed a total of 435 patients with OC in the craniofacial region. The most frequently affected site was the mandibular condyle (88.3%), followed by the coronoid process (8.7%) [3]. Only 4 cases have been reported previously involving the mandibular symphysis region [4, 5], and 5 cases were reported involving the mandibular angle region [6], and only 1 case has been reported involving the ramus of the mandible [7]. Herein, we report an extremely rare OC in the mandibular posterior ramus region. Moreover, the present case was unique, because never before has such a large, characteristic mushroom-shaped OC been reported at the mandibular posterior ramus region.

## 2. Case Report

**Figure 1:**
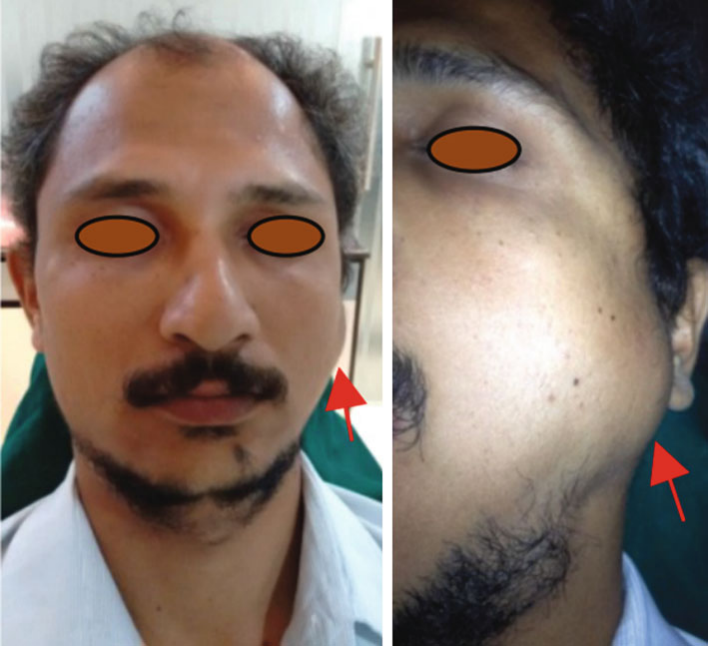
Swelling present in the front of the preauricular region on the left side of the face.

A 35-year-old male patient reported to the dental clinic with a complaint of swelling over the left middle one-third of the face for six years. History of swelling was provided as gradual in onset, slow-growing, and stable with no regression in size ever. The swelling was not associated with pain, paresthesia, discharge, or difficulty in eating. No significant history of trauma, infection, or medical conditions was reported. Neither similar swelling anywhere in the body was recorded. Facial asymmetry was present with restricted mouth opening on clinical examination, with solitary localized diffuse swelling at the preauricular region, just in front and 1 cm below the level of the left tragus of the ear. The swelling size was approximately 2 cm × 2 cm in diameter with diffuse periphery (Figure 1). The surface appeared to be regular with similar color and texture as that of the normal adjacent skin. No signs of ulcer, sinus, erythematic, or stretched skin were evident on inspection. Palpation exhibited a well-defined sessile base, fixed to the underlying bone and pinchable overlying skin. It was not tender and bony hard in consistency with no local rise in temperature. Intraoral examination was noncontributory. Based on history and clinical examination, a provisional diagnosis of peripheral osteoma was made with periosteal reactions, exostosis, peripheral osteoblastoma, and osteochondroma as a differential diagnosis.

**Figure 2:**
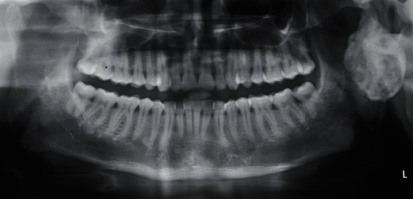
Panoramic radiograph view showed a well-defined radiopacity located at the left posterior ramus of the mandible region.

**Figure 3:**
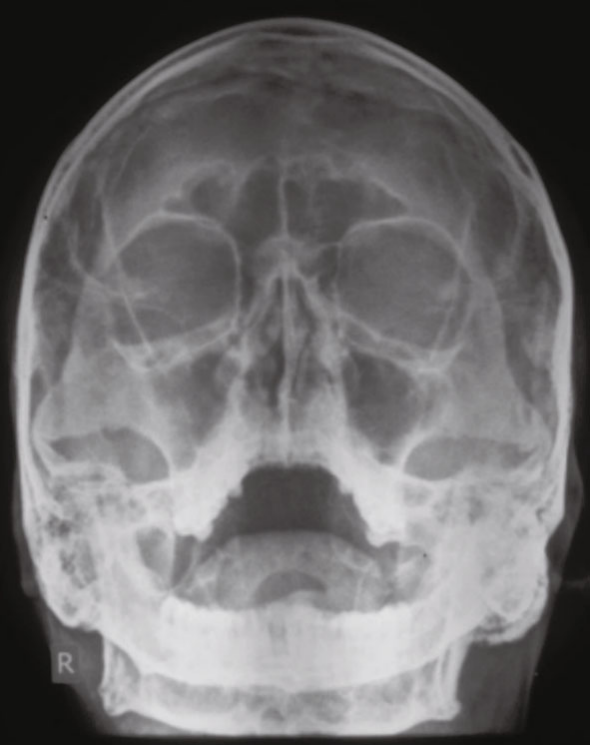
Paranasal sinus view showed a well-defined radiopacity located at the left posterior ramus of the mandible region.

**Figure 4:**
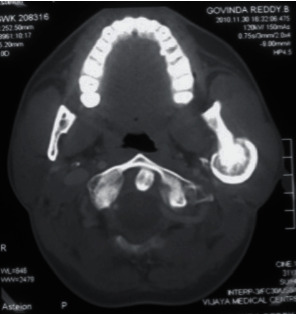
Computed tomography axial section showed a well-defined osseous bony outgrowth arising from the posterior edge of the left ramus of the mandible. The lesion showed central medullary with sclerosed cortical bone at the periphery, continuing with the condyle leading to mild elongation and giving a mushroom-shaped appearance on the axial section.

**Figure 5:**
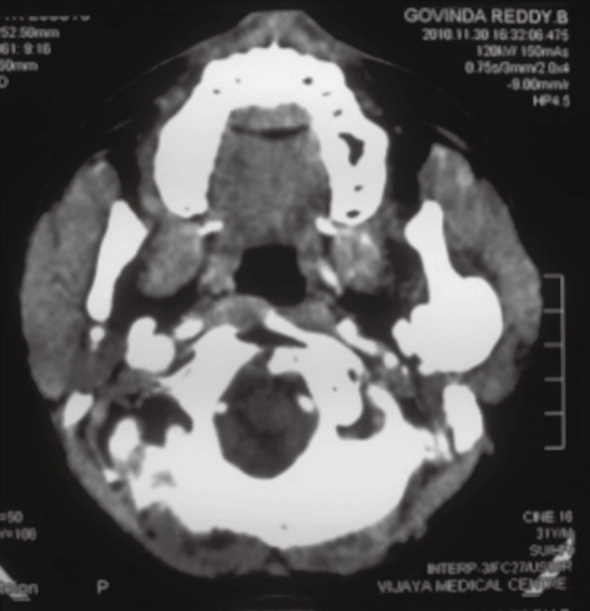
Magnetic resonance imaging scan showed continuity between the medullary cavity of the mandible and the outgrowth. It is isointense on T1 W images and hyperintense on T2 W images with the peripheral hypointense rim on the T1 W and T2 W images; these features are suggestive of the cartilage cap with mineralization.

Panoramic radiograph and paranasal sinus radiograph showed a well-defined radiopacity located at the left posterior ramus of the mandible region (Figures 2 and 3). The axial section of CT showed a well-defined osseous bony outgrowth arising from the posterior edge of the left ramus of the mandible measuring about 3.4 × 2.9 × 3.6 cm (anteroposterior × transverse × craniocaudal planes). The growth showed central medullary with sclerosed cortical bone at the periphery, continuing with the condyle leading to mild elongation and giving a mushroom-shaped appearance on the axial section (Figure 4). MRI scan showed continuity between the medullary cavity of the mandible and the outgrowth. It was isointense on T1 W images and hyperintense on T2 W images with the peripheral hypointense rim on the T1 W and T2 W images. These features were suggestive of the cartilage cap with mineralization. Mass effect was present over the facial nerve, after its existence from the stylomandibular foramen that led to the lateral displacement of the facial nerve (Figure 5). Based on the clinical and radiological features, it was suggested as osteochondroma with lateral displacement of the facial nerve.

**Figure 6:**
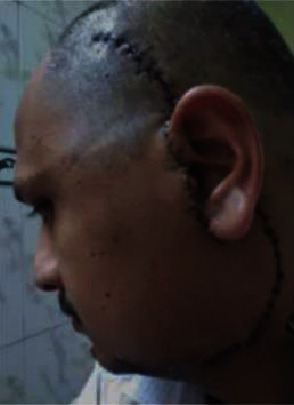
Surgical exploration of the growth was performed through the Alkyth-Brameley preauricular and submandibular approach.

**Figure 7:**
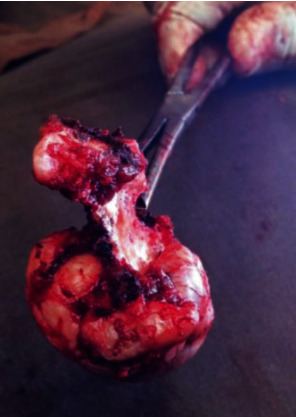
On gross examination the lesion exhibited a uniformly dense ossified area with few irregular soft tissues at the periphery.

**Figure 8:**
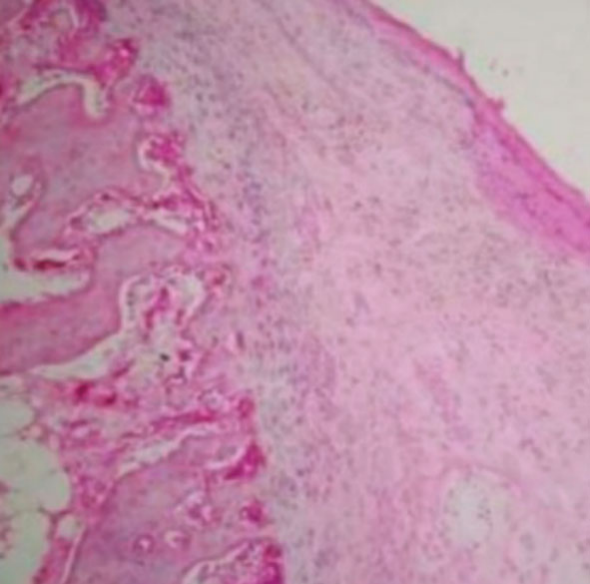
H&E staining showed fibrocollagenous tissue overlying cartilaginous islands merging into bone trabeculae.

**Figure 9:**
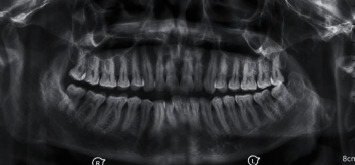
Postoperative panoramic radiography showing excision of the lesion and free of recurrence 1 year after surgery.

Surgical exploration of the growth was performed through the Alkyth-Brameley preauricular and submandibular approach (Figure 6) and osteotomy, with the cut made at the angle of the mandible. After complete surgical excision of the growth, it exhibited a uniformly dense ossified area with few irregular soft tissues at the periphery (Figure 7). The tissue section was decalcified, and H&E staining showed fibrocollagenous tissue overlying cartilaginous islands merging into bone trabeculae separated by fat marrow (Figure 8). All these features were suggestive of osteochondroma. The patient was kept under observation and was free of recurrence until one year after surgery (Figure 9).

## 3. Discussion

WHO defined OC as cartilage-capped bony projection arising from the external surface of bone containing a marrow cavity that is continuous with that of the underlying bone [8]. Various hypotheses were postulated for the pathogenesis of osteochondroma. Virchow was the first to speak about bony osteochondroma in 1891. Keith suggested that an imaginary defect in the thin cortical bone results in spillage of epiphyseal cells into the metaphysic region, and this commonly occurs in tendon insertion sites. These sites have focal accumulation of embryonic connective tissue with cartilaginous potential. Langenskiold postulated that osteochondroma occurs when limited portions of the undifferentiated cell layer of the growth cartilage are displaced toward the metaphysic peripherally. Lichtenstein and Muller's theory suggested that the periosteum had an inherent potential to form chondroblasts and osteoblasts. Thus, an osteochondroma may arise due to induced or spontaneous metaplasia of the periosteum [7, 9].

Osteochondromas are very rare in the maxillofacial region. In 1899, Jacob was the first to explain an OC of the coronoid process; for this reason, this condition has been named “Jacob's disease” [6]. In the craniofacial skeleton, it more commonly occurs around the temporomandibular joint because the region from the mandibular lingula to the anterior process of the malleus is derived from the part of Meckel's cartilage not replaced by mandibular bone, and those remnants of this embryonic tissue may persist in that region [7]. In the mental region, one or two cartilages ossify and form mental ossicles, which become incorporated into the intramembranous bone. Residues of these cartilage precursors in the symphysis appear to form OC of this region. According to cytogenetic analyses EXT1 (8q24,1) and EXT2 (11P11.2-12), loci may be involved in the pathogenesis of hereditary osteochondromas [5, 10].

They have been reported in varied locations of the craniofacial region, where the most common sites are the coronoid process and the condyle [6]. It is a slow-growing, most-of-the-time asymptomatic tumor. Sometimes, the patient may present with facial asymmetry, malocclusion, pain, and hypomobility of the temporomandibular joint. The mouth opening is in the normal range in most of the cases because of the pseudoarticulation around the mass [11].

Panoramic radiography is the primary radiologic examination that is required for diagnosis. An OC appears as a stalk or a flat protuberance emerging from the surface of the bone. On occasion, it ends up as a hook-like formation. Its margins are usually well defined, although the tumor seems to be continuous with the cortex of the bone. A usual finding is that of radiopaque calcified flakes or linear interruptions inside the cartilaginous component of the OC. Panoramic radiography helps in diagnosing the lesion as benign or malignant. Sometimes, an osteochondroma turns into osteosarcoma indicating enlargement of the tumor and the irregularity of its margins. Multiplication of the ossifications, pain, scattered calcifications, and a coexisting radiopaque soft tissue mass may suggest a sarcomatous transformation. In addition, the presence of periosteal reaction with lobulated margins may be present in osteosarcoma [4, 9].

CT and cone beam computed tomography (CBCT) are accurate methods for depicting OC. They can show the bony lesion in detail, as well as show the presence of calcifications and thickness of the cartilaginous cap. But CT and CBCT cannot estimate the metabolic activity, a serious indication of malignancy of any tumor. A scintigraphic method is being used to examine the metabolic activity of the tumor. A poor metabolic activity is only present in benign lesions. Ultrasound radiography helps to examine the cartilaginous cap of the OC as a hypoechoic area above the cortex of the relevant bone [3, 9].

MRI can know the exact morphology of a tumor and any compress over the surrounding structures. MRI is used to verify the continuity of the growth with the cortex of the affected bone. MRI shows a well-defined irregular lesion showing signal intensity similar to that of bone marrow in the center, surrounded by an iso- to hyperintense rim with few hypointense areas predominantly in the posterior aspect suggestive of the cartilage cap. The cartilaginous cap, as it is rich in water, presents a high signal on T2-weighted MRI and a low one on T1-weighted imaging. T2-weighted MRI is preferable because it provides better differentiation of signal intensities, and differentiation in the signaling of a nerve may suggest its suppression or damage. Woertler et al. indicated that the cartilage cap thickness exceeding 2 cm in adults and 3 cm in children should raise the suspicion of malignant transformation. Chondrosarcoma is also characterized by low T1 signal after intravenous contrast infusion (gadolinium), something that is rarely recorded in a benign cartilaginous tumor. So MRI is the gold standard technique for detecting a malignant transformation [6, 9].

Regardless of the etiology, the goal of treatment is complete resection of the lesion and recovery of acceptable mouth opening. After surgical excision, the recurrence rate is found to be 2% in solitary osteochondroma cases all over the body. No signs of recurrence were found in our case until now. Malignant change may occur in about 5% of cases with multiple hereditary lesions, but solitary osteochondromas have a 1% risk of malignant transformation [11].

## 4. Conclusion

Craniofacial osteochondroma, in the mandibular posterior ramus region, is extremely a rare entity. Panoramic radiography at best can be considered a screening modality in the detection of these lesions. Because of the characteristic radiographic appearance of mandibular osteochondroma, CT and MRI provide a valuable tool to assist in evaluation and treatment planning. The recommended treatment of choice for symptomatic osteochondromas is surgical resection and follow-up because it may transform into osteosarcomas.
